# Supplementary material for: Can expected error costs justify testing a hypothesis at multiple alpha levels rather than searching for an elusive optimal alpha?
Source: PLoS One. 2024 Sep 25;19(9):e0304675. doi: 10.1371/journal.pone.0304675 (PMC11424007; doi:10.1371/journal.pone.0304675)
Supplement: S1 File — (PDF) [file pone.0304675.s001.pdf]

S2: Total cost of the multi-alpha test as a weighted average of the costs of the individual tests

If the relative cost of Type I to Type II errors is independent of test level in a multi-alpha test, then the total cost is a weighted average of the single-level test costs. We show why this is so, and look at the special case when costs at different alpha levels are proportional to their surprisal value (Rafi & Greenland, 2020).

Consider a scenario in which true and false hypotheses are dichotomously distributed, with d the difference between the two values in a particular study. Let alpha levels α_m, costs $C_i(m)$ and cost differences $\Delta C_i(m)$ be defined as in section 2 of the main paper, for m = 1, …, k and i = 0,1. Suppose that for each m $C_1(m) = rC_0(m)$ where r is independent of m. Then $\Delta C_1(m) = r\Delta C_0(m)$ and, from eqn. (6) in the main paper,

$$\omega = \sum_{m=1,k} \Delta C_0(m)\big((1-P)\alpha_m + rP\beta(d,\alpha_m)\big). \qquad \text{(S2.1)}$$

Since $\sum_m \Delta C_0(m) \equiv C_0(k)$, with these assumptions the total cost of the multi-alpha test is a weighted average of the costs of the individual tests given in (1) after setting C_0 to $C_0(k)$ and C_1 to $C_1(k) = rC_0$. Specifically,

$$\omega = \sum_{m=1,k} \left(\frac{\Delta C_0(m)}{\sum_m \Delta C_0(m)}\right) \omega(d,\alpha_m),. \qquad \text{(S2.2)}$$

where $\omega(d,\alpha_m)$ is as in eqn. (1) in the main paper.

We applied the notion of the surprisal value of a P-value, introduced by Razi and Greenland (2020), to define the surprisal value $\log_2(\alpha_m)$ of a test being rejected at level α_m. Now suppose there are constants C, C', such that

$$C_0(m) = -C\log_2(\alpha_m),\ C_1(m) = -C'\log_2(\alpha_m) \equiv (\tfrac{C'}{C})C_0(m), m = 1, \dots, k. \qquad \text{(S2.3)}$$

Then, from the preceding argument, the total cost of the multi-alpha test is a weighted average of the costs of the individual tests. Substitute $\Delta C_0(m) = C(\log_2(\alpha_m) - \log_2(\alpha_{m-1}))$ and $\sum_m \Delta C_0(m) = C_0(k) = C\log_2(\alpha_k)$ in S2.2. Then the multi-alpha test costs are a weighted sum of the costs of the component tests with weights defined in term of surprisal values:

$$\omega = \sum_{m=1,k} \left(\frac{\log_2(\alpha_m) - \log_2(\alpha_{m-1})}{\log_2(\alpha_k)}\right) \omega(d,\alpha_m). \qquad \text{(S2.4)}$$

This analysis carries over to the scenario in which effect size e has a continuous distribution $p(e)$ and costs may be functions of e. Suppose the relationship in S2.3 holds for each effect size, so that, for example, $C_0(m;e) = -C(e)\log_2(\alpha_m) = \frac{C_0(k;e)\log_2(\alpha_m)}{\log_2(\alpha_k)}$. Let

$$\varpi(p, \alpha_m) = \int_{e \in E} \Delta C_0(m; e)\beta_0(e, \alpha_m)p(e)\, de \ + \int_{e \in R-E} \Delta C_1(m; e)\beta_1(e, \alpha_m)p(e)de. \quad \text{(S2.5)}$$

Substituting into (7) in the main paper and applying (2) gives the resulting expression for ϖ as

$$\varpi = \sum_{m=1,k} \left(\frac{(\log_2(\alpha_m) - \log_2(\alpha_{m-1}))}{\log_2(\alpha_k)} \right) \varpi(p, \alpha_m). \quad \text{(S2.6)}$$

Note that if costs are known and Type II error costs are proportional to Type I costs, alpha levels can be chosen to ensure that the relationships in (S2.3) hold. One alpha can be chosen arbitrarily: say, $\alpha_1 = 0.05$. Then from (S2.3) $\log_2(\alpha_m) = \log_2(\alpha_1) \frac{C_0(m)}{C_0(1)}$ and hence $\alpha_m = 2^{\log_2(\alpha_1) C_0(m)/C_0(1)}$. If the Type I error cost of decision $D(m)$ is twice that of decision $D(1)$, then α_m should be set to 0.0025 according to this formula.

Conversely, if the levels in a multi-alpha test are set (for instance, if levels 0.05, 0.01 and 0.001 are set by default), the relationship can be used to help determine appropriate decisions. Suppose decision $D(m)$ is to apply a new treatment to a population of size $q(m)$, where $q(m)$ increases with *m.* Assuming the populations have similar characteristics, costs are then proportional to the population size, so that $\frac{C_0(m)}{C_0(1)} = \frac{q(m)}{q(1)}$.

Assuming costs are related to surprisal values as in (S2.3), then $\frac{C_0(m)}{C_0(1)} = \frac{\log_2(\alpha_m)}{\log_2(\alpha_1)}$ and hence

$$q(m) = q(1) \frac{\log_2(\alpha_m)}{\log_2(\alpha_1)}. \quad \text{(S2.7)}$$

Thus, if the planned outcome from a significant result in a standard test at alpha level 0.05 was to treat a population of 1000, then a significant result at alpha level 0.001 would suggest treating a population of 1000×10/4.3 or just over 2300.

Reference:

Rafi Z, Greenland S. Semantic and cognitive tools to aid statistical science: replace confidence and significance by compatibility and surprise. BMC Med Res Methodol. 2020; 20, 244. doi:10.1186/s12874-020-01105-9
