# Supplementary material for: Can expected error costs justify testing a hypothesis at multiple alpha levels rather than searching for an elusive optimal alpha?
Source: PLoS One. 2024 Sep 25;19(9):e0304675. doi: 10.1371/journal.pone.0304675 (PMC11424007; doi:10.1371/journal.pone.0304675)
Supplement: S2 File — (PDF) [file pone.0304675.s002.pdf]

# *S3: Averaged results from simulations with random costs*

To further illustrate the impacts of cost and prevalence assumptions, we simulated one-sided $t$-tests with randomly generated costs. The hypothesis of interest is that the standardized effect size is at least $M$. Here, we report averaged results over 2000 runs at each parameter setting.

In applying eqn. (6) in the main paper, each cost difference $\Delta C_0(m), m = 1, \dots, k$ is randomly drawn from a uniform distribution on the interval [0, 100]. This ensures that on each run $C_0(m) \geq C_0(m-1)$ but, unlike the examples in the paper, does not relate the cost differences to differences in surprisal values. Corresponding values for $\Delta C_1(m)$ are randomly drawn from the interval [0, 25] so that, on average, Type I costs are four times more expensive.

Tables S3.1 and S3.2 report averaged results with standard deviations shown in brackets—because costs vary over relatively large ranges, the standard deviations are relatively large. The lowest cost from the single-level tests is shown in bold. Optimal costs are the average of the lowest cost at each run and are obtained at different test levels in different runs.

*Table S3.1. Average expected total error costs in research scenarios with dichotomous distributions of effect sizes.*

(a)

| | $\alpha_1$=0.025 | $\alpha_2$=0.0025 | multi-level | optimal |
|---|---|---|---|---|
| *d* =0.15 | | | | |
| *P*=0.5, *n*=24 | 12.8 (4.8) | **12.3 (5.0)** | 12.6 (4.9) | 11.9 (4.9) |
| *P*=0.5, *n*=192 | **11.4 (4.3)** | 12.0 (5.0) | 11.7 (4.7) | 10.6 (4.2) |
| *P*=0.1, *n*=24 | 4.6 (1.3) | **2.7 (1.0)** | 3.6 (1.2) | 2.5 (1.0) |
| *P*=0.1, *n*=192 | 4.4 (1.2) | **2.7 (1.0)** | 3.5 (1.1) | 2.5 (1.0) |
| *d* =0.5 | | | | |
| *P*=0.5, *n*=24 | **11.2 (4.1)** | 12.2 (4.9) | 11.7 (4.5) | 10.3 (3.8) |
| *P*=0.5, *n*=192 | **2.1 (0.6)** | 3.5 (1.3) | 2.8 (1.1) | 1.9 (0.6) |
| *P*=0.1, *n*=24 | 4.2 (1.2) | **2.6 (1.0)** | 3.4 (1.1) | 2.5 (1.0) |
| *P*=0.1, *n*=192 | 2.4 (0.9) | **0.9 (0.3)** | 1.7 (0.7) | 0.9 (0.3) |

(b)

| | $\alpha_1$=0.025 | $\alpha_2$=0.0025 | $\alpha_3$=0.0005 | multi-level | optimal |
|---|---|---|---|---|---|
| *d* =0.15 | | | | | |
| *P*=0.5, *n*=24 | 19.7 (5.9) | 18.9 (6.1) | **18.8 (6.1)** | 19.2 (6) | 18.7 (6.1) |
| *P*=0.5, *n*=192 | **17.1 (5.1)** | 18.0 (6) | 18.4 (6.1) | 17.8 (5.8) | 16.5 (5) |
| *P*=0.1, *n*=24 | 6.9 (1.6) | 4.1 (1.2) | **3.8 (1.2)** | 5.0 (1.4) | 3.8 (1.2) |
| *P*=0.1, *n*=192 | 6.4 (1.5) | 3.9 (1.2) | **3.8 (1.2)** | 4.7 (1.3) | 3.7 (1.2) |
| *d* =0.5 | | | | | |
| *P*=0.5, *n*=24 | **16.7 (5)** | 18.1 (6) | 18.4 (6.2) | 17.7 (5.8) | 15.8 (4.7) |
| *P*=0.5, *n*=192 | **3.2 (0.7)** | 5.1 (1.7) | 8.4 (2.8) | 5.5 (2) | 2.9 (0.7) |
| *P*=0.1, *n*=24 | 6.3 (1.5) | 3.9 (1.2) | **3.8 (1.3)** | 4.7 (1.3) | 3.8 (1.3) |
| *P*=0.1, *n*=192 | 3.7 (1.1) | **1.3 (0.3)** | 1.8 (0.6) | 2.3 (0.7) | 1.3 (0.4) |

*Note that the columns representing costs of single level tests are on different scales in* (a) *and* (b).

## *Results in research scenarios with dichotomous distributions*

Table S3.1 reports costs for various settings of prevalence *P* and difference *d* between *M* and the true effect size. Sample sizes are set to either 24 or 192, giving underpowered tests when *d* is small, as would be the case when researchers make over-optimistic predictions about effect size.

Table S3.1(a) shows average multi-alpha test costs lie between the costs of the two single level tests, consistent with a weighted sum, despite the simulated cost ratios for the various test levels only being fixed on average. When hypotheses are unlikely to be true ($P$ = 0.1), testing at alpha level 0.0025 is on average the lowest cost strategy and close to optimal. In balanced environments, the reverse tends to hold, with Type II errors dominating except when both effect and sample size are small.

Table S3.1(b) gives results with the addition of test level $\alpha_3$= 0.0005. Again, the multi-alpha test costs lie between the highest and lowest of the single level tests costs. Depending on the assumptions, costs for each of the single test levels can be close to the optimum or can be considerably larger than the lowest cost. However, even when $\alpha_3$-tests are lowest cost, the difference is relatively small.

## *Results in research scenarios with continuous distributions*

Here we model zero-mean normal distributions of true effects in the research scenario. When the smallest meaningful effect *M* is also zero, the probability that effects are meaningful is always 50%. When *M* = 0.64, this probability is lower, e.g., it is 10% at standard deviation 0.5 and 26% at standard deviation 1.

The less stringent test level 0.025 almost always performs best in the scenarios reported in Table S3.2. Type II errors are likely to dominate because the probability of effect sizes satisfying the alternate hypothesis is concentrated near the smallest meaningful effect size $M$. The exception is for the case where prevalence-weighted Type II error rates are almost identical for both test levels, and so the Type I error rates largely determine the lowest cost alpha level.

Fig S3.1 seeks to explain this exception. The distribution of true effect sizes in the research scenario is shown as a zero-mean normal with standard deviation 0.5. The smallest meaningful effect size is $M$ = 0.64. Fig S1(a) is for total sample size 12 and (b) for sample size 192. The dashed curves in the upper right of each plot are Type II error rates as functions of effect size $e$ given test hypothesis $e < M$ tested at one-sided levels 0.025 and 0.0025. The Type I error rates are very low, and only the rate for the test at level 0.025 is visually distinguishable as effect sizes approaches $M$.

At the small sample size, the averaged expected total costs for both test levels are similar because both Type II error rate functions are near one whenever $p(e)$ is appreciably above zero.

With the large sample size, the sample distribution is sharply peaked, as shown Fig S3.1(b) and the Type II error rate functions at both test levels drop in the range where $p(e)$ is not close to zero. Unless the costs of Type II errors are extremely low, these errors will dominate the calculations in (7) and so total costs for the more stringent test will be higher.

*Table S3.2. Average expected total error costs in research scenarios with zero-mean normal distributions of effect sizes and with standard deviation σ as shown.*

(a)

| | $\alpha_1$=0.025 | $\alpha_2$=0.0025 | multi-level | optimal |
|---|---|---|---|---|
| *M* = 0 | | | | |
| *σ* = 0.5, *n*=24 | **10.4 (4.1)** | 11.8 (4.8) | 11.1 (4.5) | 8.5 (3.1) |
| *σ* = 0.5, *n*=192 | **5.3 (2.1)** | 7.1 (2.9) | 6.2 (2.5) | 3.6 (1.3) |
| *σ* = 1, *n*=24 | **7.4 (2.8)** | 9.6 (3.7) | 8.5 (3.3) | 5.2 (1.8) |
| *σ* = 1, *n*=192 | **2.9 (1.1)** | 4.0 (1.6) | 3.4 (1.4) | 1.9 (0.6) |
| *M*= 0.64 | | | | |
| *σ* = 0.5, *n*=24 | 2.5 (1.0) | **2.5 (1.0)** | 2.5 (1.0) | 2.4 (1.0) |
| *σ* = 0.5, *n*=192 | **1.6 (0.6)** | 2.0 (0.8) | 1.8 (0.7) | 1.4 (0.5) |
| *σ* = 1, *n*=24 | **4.6 (1.8)** | 5.6 (2.2) | 5.1 (2) | 3.8 (1.3) |
| *σ* = 1, *n*=192 | **2.1 (0.8)** | 2.8 (1.1) | 2.5 (1) | 1.5 (0.5) |

(b)

| | $\alpha_1$=0.025 | $\alpha_2$=0.0025 | $\alpha_3$=0.0005 | multi-level | optimal |
|---|---|---|---|---|---|
| *M* = 0 | | | | | |
| $\sigma$ = 0.5, *n*=24 | **15.6 (5.1)** | 17.8 (6) | 18.3 (6.2) | 17.3 (5.8) | 13.2 (3.8) |
| $\sigma$ = 0.5, *n*=192 | **8.0 (2.6)** | 10.7 (3.5) | 12.2 (4) | 10.3 (3.4) | 5.6 (1.5) |
| $\sigma$ = 1, *n*=24 | **11.0 (3.5)** | 14.3 (4.7) | 15.9 (5.2) | 13.7 (4.5) | 7.9 (2.1) |
| $\sigma$ = 1, *n*=192 | **4.3 (1.4)** | 6.0 (2) | 6.9 (2.3) | 5.7 (1.9) | 2.9 (0.8) |
| *M*= 0.64 | | | | | |
| $\sigma$ = 0.5, *n*=24 | 3.7 (1.1) | **3.7 (1.2)** | 3.7 (1.3) | 3.7 (1.2) | 3.6 (1.2) |
| $\sigma$ = 0.5, *n*=192 | **2.4 (0.8)** | 2.9 (1) | 3.2 (1) | 2.8 (0.9) | 2.1 (0.6) |
| $\sigma$ = 1, *n*=24 | **6.8 (2.2)** | 8.3 (2.7) | 8.9 (2.9) | 8.0 (2.6) | 5.8 (1.6) |
| $\sigma$ = 1, *n*=192 | **3.1 (1)** | 4.2 (1.4) | 4.8 (1.6) | 4.0 (1.4) | 2.3 (0.6) |

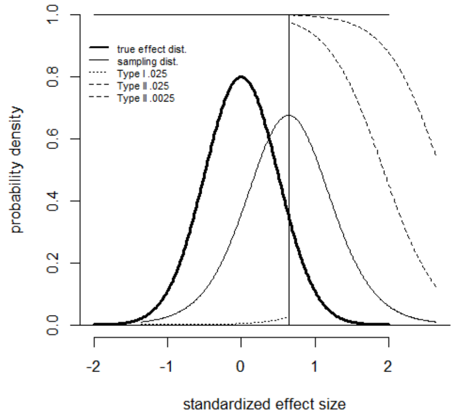


(a)

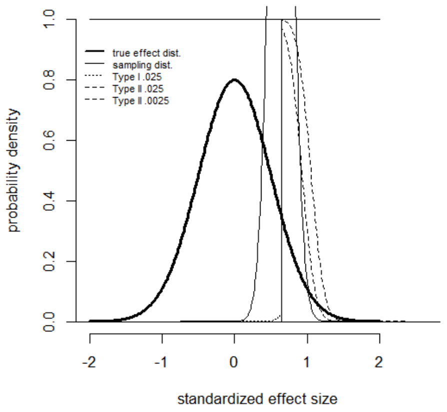


(b)

***Fig S3.1. Error rates in relation to the distribution of effects in the research scenario*** *Heavy solid curve: distribution of effect sizes in research scenario. Solid curve: sampling distribution at M =* 0.64 *given total sample sizes* 12 *(a) and* 192 *(b). The vertical lines are at the meaningful effect size boundary M.*
