# Supplementary material for: Can expected error costs justify testing a hypothesis at multiple alpha levels rather than searching for an elusive optimal alpha?
Source: PLoS One. 2024 Sep 25;19(9):e0304675. doi: 10.1371/journal.pone.0304675 (PMC11424007; doi:10.1371/journal.pone.0304675)
Supplement: S3 File — (PDF) [file pone.0304675.s003.pdf]

S4: Illustration of impact of the distribution of effect sizes in the research scenario on Type I and Type II error rates.

Here we seek to explain some findings reported in Table 2 of the main paper. Refer to section 3 for assumptions and notation, including the assumption of two-group trials with equal size groups.

Table 2 showed that the optimal one-sided test level was 0.23 when true effects were assumed to have a zero mean normal distribution with standard deviation 0.025. The vertical lines in Fig S4.1(a) are at critical values when testing the hypothesis of risk difference M at one-sided alpha levels 0.05 and 0.23. Also shown is the sample distribution at effect size -0.02. The area beneath this curve to the right of each vertical line gives the Type II error rate at the respective alpha level for this effect size; the test at level 0.05 would therefore incur very high costs for an effect of this size.

In continuous scenarios, error rates with respect to an effect size are multiplied by the probability density at that effect size to give an expected rate. Fig S4.1(b) depicts the optimistic research scenario in which treatments have more than 50% chance of being meaningful (solid curve) and the pessimistic scenario where the probability of a meaningful treatment effect is 12% (dashed curve). The test level 0.05 is close to optimal in the pessimistic scenario because Type II errors are rare.

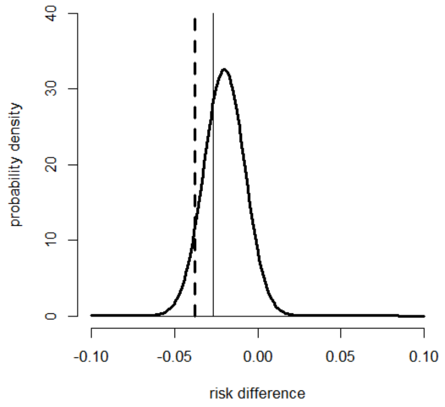

(a)

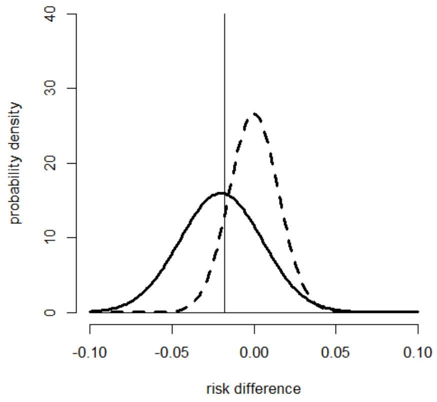

(b)

Fig S4.1. (a) Sampling distributions and critical values *The dashed (resp. solid) vertical line is at the critical value when testing for risk difference M at alpha level* 0.05 *(resp.* 0.23*) using the sampling distribution shown as dashed curve. Solid curve is the sampling distribution given true effect size –*0.02. ***(b) Research scenario distributions*** *Distribution of effect sizes in research scenarios with zero mean and standard deviation* 0.015 *(dashed curve) or mean* –0.02 *and standard deviation* 0.025 *(solid curve). Vertical line is at the risk difference M below which intervention is cost effective.*
