# Supplementary material for: Can expected error costs justify testing a hypothesis at multiple alpha levels rather than searching for an elusive optimal alpha?
Source: PLoS One. 2024 Sep 25;19(9):e0304675. doi: 10.1371/journal.pone.0304675 (PMC11424007; doi:10.1371/journal.pone.0304675)
Supplement: S4 File — (PDF) [file pone.0304675.s004.pdf]

S1: Testing one hypothesis at multiple alpha levels: theoretical foundation and indicative reporting

"Testing a hypothesis at multiple levels" is shorthand for saying that a family of hypotheses, constructed according to the procedure described below, is to be tested as described, with each family member tested at a single level. We then look at how results from such tests would be reported.

Formal construction

Formally, suppose the test hypothesis $H: e \in E$ is to be tested at levels $\alpha_1, \alpha_2, \ldots, \alpha_k$. Let R be the relevant space of effect sizes and let $R(\alpha_m)$ denote the rejection region of H for the α_m-level test.

Now take an independent parameter space A and subsets $A_m \subseteq A$ such that $A_{m'} \subseteq \mathrm{A}_m$ implies $m' = m$, where $m', m \in \{1, \ldots, k\}$. For each m, form the hypothesis ${H^A}_m: a \in A_m$. Let $R^A(\alpha_m)$ be the rejection region of ${H^A}_m$ tested at level α_m and suppose the intersection of these rejection regions is non-empty. Choose any $x \in \cap \{R^A(\alpha_m): m = 1, \ldots, k\}$. Aisbett (2023) gives two examples of parameter spaces, subsets and tests that satisfy these conditions.

Next, let **H** be the family of hypotheses $H_m: (e, a) \in (E \ \times A\ ) \cup (R \times A_m), m \in \{1, \ldots, k\}$. By construction, $H_{m'}$ implies H_m only if $m' = m$. To inject data into the extended parameter space $E \times A$, take observation *v* to the vector (v, x).

If H_m is tested at level α_m its rejection region is $R_m = R(\alpha_m) \times R^A(\alpha_m)$ (Aisbett, 2023).

It follows that the extended data lead to rejection of hypothesis H_m tested at level α_m if and only if the original data lead to rejection of H at this level. Thus, testing H at multiple levels is shorthand for saying we are testing members of the family **H** at respective levels $\alpha_1, \alpha_2, \ldots, \alpha_k$. Likewise, we will speak about H being rejected at alpha level α_m but not at level α_{m+1} as shorthand for saying that H_m is rejected but H_{m+1} is not.

As example, suppose the test hypothesis H is that a parameter d is in Δ, where Δ could be, say, the negative real numbers, and suppose data consist of a set of observations d_i, i=1,…n. Further suppose test levels are 0.005 and 0.01. To extend the parameter space we introduce a normally distributed random variable with variance 1 and unknown mean m. Form two composite hypotheses about the value of the vector (d, m), namely:

> the hypothesis that d is in Δ OR $0.005 < m < 0.01$, to be tested at level 0.01;
>
> the hypothesis that d is in Δ OR $0 < m < 0.005$, to be tested at level 0.005.

Extend each observation d_i to the vector (d_i, z) where z is in the rejection region of the test hypothesis H_m: $m > 0$ at level 0.005. Since H_m is always rejected on the extended data, the composite hypotheses above are rejected only when H is rejected on the original data set at their given test level.

Reporting

Aisbett (2023) argues that testing at multiple levels provides a bridge between those who view P-values as a continuous measure of strength of evidence against the test hypothesis, and those who apply thresholds to make dichotomous decisions. How findings of multi-level tests are reported thus has features of both reporting P-values and making decisions.

Tabular presentations

When multiple interventions are investigated in a trial, common practice is to report findings in a table with a column containing P-values. Entries in this column may be annotated with one or more asterisks (stars) depending on their values. The same convention would be used in reporting tests at multiple alpha levels. However, these levels will not necessarily correspond to the thresholds at which statistical software assigns asterisks (usually 0.05, 0.01 and 0.001). The table caption should explain the annotations as, for example, *"*** statistically significant at alpha level 0.003.*"

Text Reporting

As with single level tests, how findings from multi-level tests are reported depends on the intended audience.

Consider a trial investigating whether an intervention is as effective as current practice. The research team decides *a priori* to conduct *t*-tests at one-sided alpha levels of 0.05, 0.01 and 0.001. Analysis of the data returns a P-value of 0.03.

Formally, the finding might be reported as:

> The conclusion that the intervention was not as effective as current practice was rejected at alpha level 0.05 but was not rejected at alpha level 0.01.

Alternatively, it might be reported as:

> The test to determine if the intervention is as effective as current practice was statistically significant at alpha level 0.05 but not at alpha level 0.01.

Because the alpha levels must be reported with each finding (rather than simply assumed to be 0.05), such reporting discourages dichotomous conclusions.

It may be more meaningful to stakeholders to report how *compatible* the hypothesis of effect is with the data (Rafi & Greenland 2020). Suppose the three test levels are respectively considered to be "weak", "moderate" and "strong" in the research context. Then the finding might be reported as:

> The conjecture that the intervention would be as effective as current practice was only weakly compatible with our trial data.

For more general audiences, communication might be better served by an informal statement such as:

> Our data provided only weak evidence that the intervention is as effective as current practice.

This style of reporting is similar to the reporting of single level tests when P-values have been awarded one or more stars.

Confidence intervals

Under the duality between critical values and confidence interval (CIs), the critical values of multi-level tests correspond to CIs about the same test statistic, with upper and lower limits that are functions of the critical values. Thus, the CIs associated with multi-level tests overlap, their length increasing as the test level becomes more stringent. Fig S1.1 illustrates.

This figure was generated using the function *multilevelCI*() available at *github.com/JA090/ErrorCosts*. Parameters include test statistics, SEs, critical values and labels to describe test levels and interventions. A function to transform the summary data can also be nominated and the CIs can be oriented either horizontally or vertically.

However, any software that allows line widths, line colors or line types to be set when drawing CIs might be used to overlay CIs with different confidence levels in this manner.

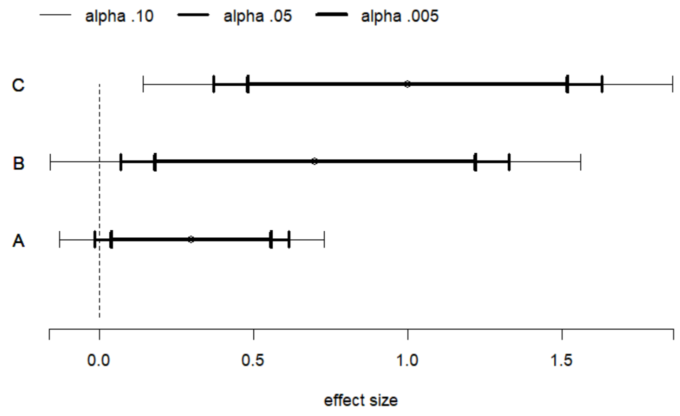

Fig S1.1. Confidence intervals corresponding to testing at multiple alpha levels. *The CIs indicate that for test* A, *the null hypothesis is rejected at alpha level* 0.10 *but not at level 0.05. For test* B, *the null hypothesis is rejected at level* 0.05 *but not at* 0.005, *while for test* C *it is rejected at level* 0.005.

Compact letter displays

In some disciplines, compact letter displays are routinely used when reporting pairwise comparisons of outcomes from multiple treatments groups. The mean+SE or a box plot for each group is annotated with one or more alphabetic characters. Groups that share a character are not significantly different under some multiple comparison test at a family-wise alpha level of 0.05.

When testing at multiple family-wise alpha levels, the compact letter displays should be reported in a table such as that in Fig S1.2, in which rows represent compact letter displays for each test level.

This figure was generated by function *boxPlotTable*(), available at *github.com/JA090/ErrorCosts*.

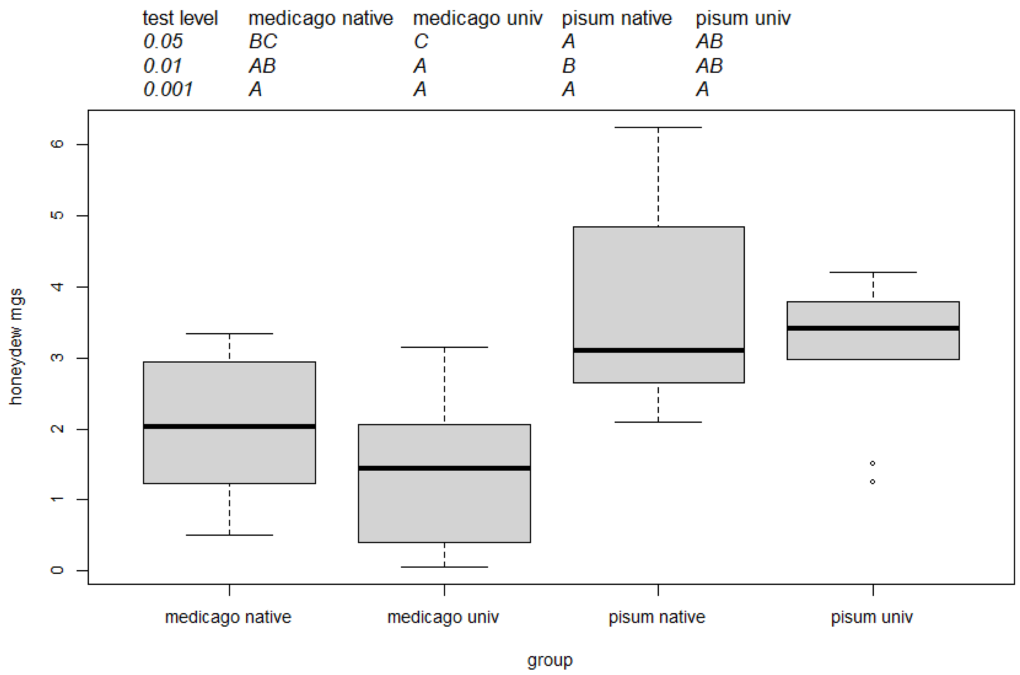

Fig S1.2. Compact letter display when a pairwise comparison test is applied at multiple family-wise alpha levels. *Table above the box plots reports findings from Tukey HSD tests. Data are from Vosteen et al (2017; Fig 2f).*

References

Aisbett J. Interpreting tests of a hypothesis at multiple alpha levels within a Neyman–Pearson framework. Statistics & Probability Letters. 2023; 201 doi:10.1016/j.spl.2023.109899

Rafi Z, Greenland S. Semantic and cognitive tools to aid statistical science: replace confidence and significance by compatibility and surprise. BMC Med Res Methodol. 2020; 20, 244. doi:10.1186/s12874-020-01105-9

Vosteen I, Gershenzon J, Grit K. Data from: Hoverfly preference for high honeydew amounts creates enemy-free space for aphids colonizing novel host plants [Dataset]. Dryad. 2017; doi.org/10.5061/dryad.37972
