# Supplementary material for: Can expected error costs justify testing a hypothesis at multiple alpha levels rather than searching for an elusive optimal alpha?
Source: PLoS One. 2024 Sep 25;19(9):e0304675. doi: 10.1371/journal.pone.0304675 (PMC11424007; doi:10.1371/journal.pone.0304675)
Supplement: S5 File — (DOCX) [file pone.0304675.s005.docx]

S5: R code to produce all Tables and Figures: See *github.com/JA090/ErrorCosts.*
