# Supplementary material for: Can expected error costs justify testing a hypothesis at multiple alpha levels rather than searching for an elusive optimal alpha?
Source: PLoS One. 2024 Sep 25;19(9):e0304675. doi: 10.1371/journal.pone.0304675 (PMC11424007; doi:10.1371/journal.pone.0304675)
Supplement: Attachment — Submitted filename: Aisbett Plos 1 Response to Reviewers.docx [file pone.0304675.s006.docx]

Stephan Leitner	22 February 2024
Academic Editor

PLOS ONE

Thank you and the referees for the valuable feedback which I have had pleasure in acting upon. My response to each suggestion is detailed below.

**Reviewer 1 comments and author responses.**

*Conduct empirical studies or simulations to demonstrate the practical implementation and advantages of the proposed method in various research contexts.*

The Discussion now suggests empirical studies in various research contexts as necessary future work. (This follows the advice of the Associate Editor on how to address the comment. Colleagues and I are currently working on applying the approach to studies into performance enhancement in sports science and to the management of invasive plant species.)

*Clarity in reporting: Provide clear guidelines on how to report and interpret results from multi-level testing to avoid potential confusion or misinterpretation.*

A new section in Supplement 1 covers reporting of multi-level tests, including confidence intervals and compact letter displays, with supporting R code. (It was hard to see where to address this important recommendation in the main paper without losing the focus on error costs.)

*Consider alternative approaches: Maier and Lakens (2022) also suggest a Bayesian-frequentist compromise to circumvent the problem of having to deal with ill-defined error costs, assumptions of hypothesis prevalence and power estimation. This approach was recently expanded to regression type models by Wulff and Taylor (2023). The paper could benefit from a discussion comparing the proposed approach to this Bayesian-frequentist approach.*

The Discussion now suggests setting multiple alpha levels within this Bayesian-inspired framework, using the associated R code. The idea is to take boundaries of the relative evidence categories as default BFs, which correspond to “default” alphas that are functions of sample size. Relative costs of errors would still have to be considered if setting BFs rather than using defaults. (Thanks for the Wulff & Taylor reference.)

**Reviewer 2 comments and author responses.**

 *I think one point that is not sufficiently addressed is that interpreting p-values as strength of evidence if not part of the classical Neyman-Pearson view. The author acknowledges that on page 3, but then only gives a very brief description of how Aisbett (2023) overcomes this problem. I think this section would benefit from a more detailed explanation of how the proposed approach relates to significance testing as thought of by Neyman and Pearson.*

This comment has been addressed in two ways. Firstly, the explanation of the construction in the main paper has been expanded, and an example has been added to Supplement 1. This hopefully clarifies the treatment of the multi-level test as a multiple hypothesis testing problem sitting within the Neyman-Pearson framework. Secondly, findings are now said to be explicitly tied to “test level” instead of “evidence level”.

*Often it is difficult to envision what the multiple decisions based on the different alpha levels would be. Scientists may often decide to either make a claim or not, or in practice, policymakers may decide to either roll out a new drug or not. I think the paper would benefit from more concrete examples that describe the different decisions that would be made as a function of the alpha interval within which the test was significant.*

An example from veterinary science is now used throughout Section 2 to illustrate the various concepts. These examples include multiple decisions, their associated costs, and how costs should link to the multiple test alpha levels.

*The paper starts out very theoretical and only gives examples towards the end of the text. I think it would improve readability, especially for experimental researchers, who are the ultimate target audience a lot if the concepts were explained alongside examples rather than putting all the theory first and examples in the second part*.

This excellent suggestion has been addressed using the veterinary science example—chosen because, regardless of their discipline, many researchers have a personal interest in pets and pet food.

*Paragraph two in the introduction suggests that the probability of the hypothesis being true plays no role in Mudge et al or Maier & Lakens; however, this information is actually also needed to optimise the weighted error rates.*

The references have been moved to after the second sentence to make it clearer they refer to both local and global definitions. (Although the global definition in Mudge et al that Maier & Lakens cite has to be extracted from the supplementary R code.)

*In Equation 3 a bracket seems to be missing.*

Corrected, thank you.

*The title suggests that the paper is in strong opposition to previous work on optimizing alpha levels; however, when reading the paper, it becomes clear that it is mostly an extension of previous ideas to the multiple alphas and distributions of effect sizes case. I would probably focus the title more on this element (e.g., something like “Don’t put all our eggs in one basket”).*

Revised title: “Can expected error costs justify testing a hypothesis at multiple alpha levels rather than searching for an elusive optimal alpha?”

Janet Aisbett	[janet.aisbett@meraglim.com](mailto:janet.aisbett@meraglim.com)
