# Supplementary material for: Can expected error costs justify testing a hypothesis at multiple alpha levels rather than searching for an elusive optimal alpha?
Source: PLoS One. 2024 Sep 25;19(9):e0304675. doi: 10.1371/journal.pone.0304675 (PMC11424007; doi:10.1371/journal.pone.0304675)
Supplement: Attachment — Submitted filename: response to PLOS 1 round 2.docx [file pone.0304675.s007.docx]

Stephan Leitner	5 May 2024
Academic Editor

PLOS ONE

Thank you for nominating a third referee who has again provided valuable feedback. My responses to each suggestion are detailed below.

Following this referee’s concern at possible confusion with multilevel models, the term “multi-level” has been replaced throughout the manuscript by “multi-alpha”. This new nomenclature is also used in the responses below.

**Reviewer 3 comments follow in italics, with responses in plain text.**

1. a. *In line 190, R – E is introduced as the subset of “true” effects, but E is the subset of the effect sizes for true hypotheses and R the full domain. The term “test hypothesis” being synonymous to the null hypothesis is not a convention in some fields of research (e.g. Psychology). This should be better clarified. In the next paragraph the authors write “… when true effects are further away from R – E, …”, which seems to contradict the previous statements. It would imply that true effects are somehow “further away” from true effects. I suspect that the authors have made at least one accidental flip somewhere in the wording, or, if there is no mistake, need to rewrite the paragraph for understandability.*

R – E is now defined less ambiguously as the subset of “effects for which the research hypothesis is true”.

The term “test hypothesis” is now explained when it is first introduced (paragraph 3 of the Introduction) as the “hypothesis that the researcher hopes to reject—typically the null hypothesis of no effect.” It is defined again when specifying the subset E as effects for which the test hypothesis is true.

The dog diet example is now used to illustrate what it means to be “further away” from the set R – E, with a negative weight loss (dogs on the diet actually put on weight) being distant from the set of losses greater than the smallest meaningful loss 0.1.

b*. In Equation 3, it is unclear what values the index m of the sum should be able to take. This should be spelled out in the notation. Using all the values given before (0, 1, 2, …, k in lines 215f) does not work, because then C_0(m-1) would not exist for m = 0 and \alpha_0 is undefined. Furthermore, the second equality cannot be reached by rearrangement, as the authors claimed, because the final closing brackets are misplaced (they belong in front of the \alpha_m). The Equation seems correct when assuming that m can take the values 1, 2, …, k, moving the bracket, and taking the additional assumptions into account. Even then, the transformation from the left side to the right side are non-trivial (to me). For this reason, it might be worthwhile to add a derivation in the appendix or supplement, or at least expand the explanation of the equation. This erroneous equation has cost me a disproportionate amount of reviewing time.*

Apologies for the incorrect bracket positioning in eqn. 3. The range has been added to both summations. In addition, the intermediary steps that follow from the definitions $C_{0}\left( 0 \right)=0, \alpha_{k+1}=0$ are given after the equation, i.e., $\sum_{m=1,\ldots,k} C_{0}(m)\alpha_{m+1}=\sum_{m=1,\ldots,k-1} C_{0}(m)\alpha_{m+1}\equiv\sum_{m=2,\ldots,k} C_{0}\left( m-1 \right)\alpha_{m}=\sum_{m=1,\ldots,k} C_{0}\left( m-1 \right)\alpha_{m}.$

*c. Supplement 2: In Equation S2.1 the variable “d” is included without proper introduction. The effect size notation in the main text suggests to me that “e” would be correct here because d is only used for specific effect sizes in the examples (e.g. Eq. 10). In Equation S2.6 “p” could be introduced more approachably.*

*d* is now defined and the reader is referred to the main paper for the definitions of other variables appearing in (S2.1). *p* is now defined as a probability density function and the expected total error cost $\varpi\left( p,\alpha_{m} \right)$ is defined in a new (S2.5).

2. *Individual p-values can be highly unreliable indicators of the strength of the evidence (see e.g., Fig. 1 in Dienes (2014)). On the one hand, the authors provide a mechanism of caution by using multiple alpha-levels. On the other hand, they only implicitly address the issue that there is not only uncertainty that is quantified by the p-value, but also uncertainty in the random draw of the p-value itself. In my view, this should be discussed as a limitation explicitly. It seems especially relevant in one-off applications, in which researchers might develop a false confidence in the exact p-value when using the suggested approach. Only when considering the average error cost across many repetitions does this become a negligible detail that is implicitly treated in the calculations by the authors.*

The following cautionary paragraph has been inserted before the last paragraph of the Discussion:

Whatever levels are chosen, testing at multiple alphas is subject to the qualifications needed for any application of P-values, which only indicate the degree to which data are unusual *if* the test hypothesis together with all other modelling assumptions are correct. Data from a repeat of the trial may still, with the same assumptions and tests, yield a different conclusion.

3. *Despite discussing decision making in a state of uncertain evidence, the authors do not consider collecting more data as a choice alternative. Maybe this is more obvious in the Bayesian framework, where there are Bayes-factors that are understood as inconclusive and additional data can be collected at will to arrive at a more conclusive result. Within the logic of the current manuscript, the state of inconclusiveness is also explicitly discussed, with partial investment as a suggested typical strategy (e.g. in lines 243 ff.). Given that the authors already consider a palette of choices based on the clarity of the evidence, collecting more data should be discussed as an obvious alternative to minimize error costs. This seems highly relevant if data collection is comparatively cheap. (Remark: The parallel to Bayesian hypothesis testing may generally be interesting to explore. There, researchers may automatically tend towards collecting more data when the Bayes factor is very inconclusive regarding the hypotheses.)*

The excellent suggestion of collecting more data replaces the decision to “not publish” that the reviewer pointed out was both unnecessary and controversial in their minor comment *line 299*.

4. *A key limitation of the authors’ approach to decision making seems to be that the computation of the cost of errors in empirical hypothesis testing at the time of hypothesis testing may be impossible in many areas. For this reason, it may also be impossible to generate a sensible set of decision alternatives and alpha levels. In other situations, it may not be clear what the potential decisions are. The value of foundational research, for example, lies largely in its role as groundwork for future research and innovation, which is, by definition, unknown at the time. To some degree all research has this knowledge-generating value. More generally it is unclear how (or if) the idea of a set of decisions representing various levels of investment can be applied to general research in the empirical sciences (see also related comment by Reviewer 2 from the first round of reviews). For example, it is unclear why several alpha levels would be needed in a study that has no specific decisions bound to it, yet, and could instead just report the p-value. The authors could more clearly outline under which conditions their approach to hypothesis testing is practical and under which conditions it is rather infeasible or may even be misleading.*

(See also the Response to 5).

The difficulty of determining costs is agreed! The examples and simulations aimed to show how expected total costs from multi-alpha tests at pre-set alphas were acceptable if not optimal. The Discussion may have muddied the waters by describing two strategies for choosing alphas that require estimating some costs and decisions.

The first strategy of taking high and low “optimal” alphas calculated using a range of prevalence models has been expanded to allow for a range of cost models.

The second strategy based on stakeholders determining decisions and costs is now explicitly said to be “appropriate to applied research.”

The third strategy of using default alpha values does not depend on estimating costs and has been highlighted by:

(i) expanding the conclusion of the Abstract as follows:

Testing at multiple default thresholds removes the need to formally identify decisions, or to model costs and prevalence as required in optimization approaches. Although total expected error costs with this approach will not be optimal, our results suggest they may be lower, on average, than when “optimal” test levels are based on mis-specified models.

(ii) reorganizing the Discussion so that it is the last of the three strategies to be presented.

The Introduction pointed out that an advantage of testing at multiple levels over simply reporting P-values is the *a priori* attention given to the relative importance of Type I and Type II errors, and to sample size. That is, even though every researcher should report raw P-values, at the design stage of any study it is essential to think about whether the study has the power to detect potential effect sizes. I think this applies broadly.

4a. *Operationalizing the costs of non-treatment by only looking at hospitalization without ICU seems to oversimplify the scenario in a dangerous way. This is besides the main point of the study, but maybe an indicator of how difficult it is to determine the costs of decision errors.*

The source paper on Covid treatment costs had data on mechanical ventilation as another layer above ICU, so a fuller analysis would have made the example unnecessarily complicated. That said, it is true that estimating costs *is* always going to be complicated.

5. *Only beginning in line 238, the authors clarify that the multiple hypothesis tests are linked to different decisions, and what these decisions could be. This may not be obvious to anyone who first reads this manuscript. Second, they do not state where the intended reduction in error costs comes from in the introduction. In essence, the mechanism behind their approach remains unnamed and undescribed until it is introduced slowly in technical detail. In general, a brief description of the main mechanism and key idea is missing. The introduction could be more straightforward towards these key points to summarize the key idea. Similarly, the beginning of the discussion could focus on the main takeaways earlier instead of first inserting a recollection of the findings by Neves et al. (2020). For example, what I would consider to be one of the main takeaways (lines 543f) is hidden at the end of a paragraph that starts with a different topic.*

The Abstract now explains “Error costs are tied to decisions, with different decisions assumed for each of the potential outcomes in the multi-alpha level case.”

The paragraph in the Introduction concerning losses/payoffs in relation to test levels has been clarified and expanded to read:

Thus, rather than a statistical test leading to dichotomous decisions, there may be a range of decisions, each associated with different error costs. A small P-value may be interpreted as strong evidence that triggers a decision bringing a large positive payoff when the finding is correct and a large loss when the finding is incorrect as compared with the payoff when no evidence level is reported.

The paragraph near the start of the Discussion on Neves et al (2022) has been removed.

**Responses to specific comments**

*line 53: There is a typo in the reference resulting in an impossible publication year.*

Changed to 2019.

*line 108ff: The term “multi-level” might mislead readers to confuse it with multi-level models, especially if the terminology is widely adopted in future literature or even applied to multi-level models.*

Changed to “multi-alpha” tests throughout.

*line 299: The reader may get the (wrong) impression that the authors imply that a self-censoring version of the publication bias is a non-problematic, standard decision. In any case, the decision to selective not publish null results produces a bias in the literature and therefore has a cost. This topic could be easily avoided since it adds little to the content of the manuscript.*

Changed as described in response to comment 3.

*Lines 312ff: The surprisal value of a p-value and the value of the information gained in practice also depend on prior knowledge. The authors seem to assume that no prior knowledge exists and should make this explicit. That the surprisal value given by Rafi & Greenland (2020) is a good general approximation of error costs of real decisions should be justified more clearly. (Remark: At this point researchers might seriously consider the Bayesian framework as an alternative approach, due to the seamless integration of prior knowledge in prior distributions).*

The subsection linking costs to surprisal values now notes the assumption of no prior knowledge and gives an example of typical prior knowledge. (It is true that a Bayesian approach often makes more sense, but is outside the scope of this manuscript.)

Supplement S2 now shows how the relationship between costs and surprisal values can be used to set alpha levels if costs are known. Conversely, if alphas are pre-set (e.g. are defaults) and decisions concern the extent of rollout of a treatment, as in the dog food example, it shows how the relationship can be used to determine the appropriate decision for a given alpha level.

*Lines 348f: It could be stated more clearly (here and/or above) that the direct relationship between the error costs and the p-value is an explicit simplification for the design of the hypothetical example study, removing the key step of researchers choosing decision alternatives.*

Added: “This simplifying assumption implies no prior knowledge about test outcomes.”

*Lines 580ff: The authors outline a strategy to arrive at optimal alpha levels based on assumptions about costs of different decisions. In this context it seems highly relevant to me that, all else being equal (including sample size), the p-value has a (non-linear, but) deterministic relationship to the standardized effect size estimate. This is true at least for some simple scenarios, like t-tests. The p-value is a transformation of Cohen’s d in that case. Therefore, any strategy that involves alpha levels implicitly accounts for effect sizes. This might be a relevant transformation for the computation of the costs of decision strategies because they can more easily be linked to standardized effect sizes than p-values given a sample size. In general, I found this worthwhile to remind myself of. It also means that the effect size is implicitly accounted for when using the proposed approach, although it might seem neglected at first glance. Therefore, it seems important to me to point this out somewhere in the manuscript.*

I think the best potential application of this transformation may be in setting multiple default alpha levels. This sentence has therefore been added to the Discussion.

Default alpha levels might also be assigned indirectly using default effect sizes, such as the Cohen’s d values for “small”, “medium” and “large” effects, given the functional relationships between P-values and effect sizes that depend, inter alia, on sample size and estimated variance.

*Supplement 3: The equation numbers from the main text are shown as “Error! Reference source not found.” In Figure 3, the y-axes are not labeled.*

Fixed

*Supplement 4: In Figure S4.1, the y-axes are not labeled.*

Fixed

Also labelled y-axes in Figs 1a and 1c.

Janet Aisbett
